# Developing a Decision-Support Tool to Improve the Performance and Sustainability of Cow–Calf Grazing Systems Using Satellite Remote Sensing and Mechanistic Nutrition Models

**DOI:** 10.3390/ani16111675

**Published:** 2026-05-30

**Authors:** Marcia H. M. R. Fernandes, Jordan M. Adams, Joao A. R. Fernandes, Luis O. Tedeschi

**Affiliations:** Department of Animal Science, Texas A&M University, College Station, TX 77845, USA; jmadams@tamu.edu (J.M.A.); joao@tamu.edu (J.A.R.F.)

**Keywords:** cattle, grazing management, modeling

## Abstract

Sustainable cow–calf production requires balancing animal performance, economic returns, and environmental impacts under highly variable forage conditions. This study presents a conceptual model, CattleSat, whose decision-support framework integrates satellite-derived forage biomass with mechanistic ruminant nutrition models to simulate the effects of herd size and stocking strategies on animal performance, greenhouse gas emissions, and economic outcomes. A case study simulation using data from a Texas grazing system was conducted to demonstrate the application and behavior of the model under variable herd sizes. Results showed that increasing herd size reduced forage allowance, leading to decreased cow dry matter intake and, consequently, individual animal performance, particularly milk yield and weaning weight, while total calf production exhibited a curvilinear response. Economic outcomes followed similar patterns, with total net return increasing but net return per cow declining as herd size increased. Based on the assumptions and parameterization adopted in this simulation, a critical transition point was identified where system-level profitability and individual efficiency were balanced. Additionally, carbon emission intensity increased at higher stocking rates, indicating reduced environmental efficiency. Overall, forage dynamics were relevant drivers of system variability. These findings highlight the importance of adaptive, data-driven stocking strategies and demonstrate the potential of integrating remote sensing with mechanistic models to improve the sustainability of grazing systems. Future studies and model improvements should be incorporated to expand the applicability of the framework across diverse grazing systems.

## Simple Summary

The proposed CattleSat framework provides a practical decision-support tool for cow–calf producers, researchers, and land managers to optimize stocking strategies under variable forage conditions. By integrating satellite-derived forage biomass with mechanistic nutrition and economic models, the tool enables real-time or retrospective evaluation of herd size scenarios to balance productivity, profitability, and environmental performance. This approach can support adaptive grazing management, improve forage utilization efficiency, and reduce greenhouse gas emission intensity by identifying optimal stocking thresholds. The framework is particularly applicable in regions with high climatic variability, where traditional fixed stocking strategies are ineffective, and can be expanded to precision livestock farming systems that leverage remote sensing and data-driven management.

## 1. Introduction

The United States is the world’s leading beef producer [1]. However, this leadership position comes with significant responsibilities and challenges regarding production sustainability. Cow–calf operations, which form the foundation of the beef supply chain and rely predominantly on grazing systems, play a pivotal role in determining the industry’s overall environmental impact and long-term viability [2].

Sustainability refers to a system that is economically viable, socially beneficial, and environmentally responsible [3]. Sustainable grazing systems, which form the foundation of cow–calf and stocker operations worldwide, depend on the prudent use of resources to effectively balance animal requirements with available forage supply. The advancement of scientific knowledge in ruminant nutrition, coupled with sophisticated mathematical modeling, has revolutionized our understanding of livestock systems. Modern mechanistic nutrition models can accurately predict nutrient intake, digestibility, and animal performance, enabling producers to optimize both production efficiency and grazing practices for domesticated ruminants [4,5,6]. These nutrition models can be integrated into decision-support tools (DST) to predict energy expenditure and intake of grazing animals [7,8]. For instance, Tedeschi et al. [9] proposed a mathematical model to compute a cow energy efficiency index based on the metabolizable energy required for a given calf weaning weight (WW), but pointed out the limitation imposed by the lack of reliable technologies to assess both the quantity and quality of pasture forages.

Traditional methods for assessing pasture forage quantity and quality have relied heavily on manual monitoring and field experience. However, remote sensing techniques have emerged as a viable alternative to conventional field and laboratory analyses for monitoring grazing lands [10], offering a means to address the complexities of grazing systems. Satellite remote sensing provides a transformative solution by delivering real-time data on vegetation biomass, forage quality, and environmental conditions [11]. Integrating these data with mechanistic nutrition models enables producers to optimize animal performance and feed efficiency, ultimately facilitating the identification of effective management strategies and cow genotypes best suited to specific grazing ecosystems.

Nonetheless, one of the main challenges in modeling grazing systems is integrating the “animal component” with the dynamics of the “pasture component” because the intake of a grazing animal is driven not only by the nutritive value of the forage, but mainly by the pasture structure combined with its availability [12]. As a function of forage mass (FM) and stocking rate [13], forage allowance (FA) has been shown to be a powerful tool to quantify the amount of forage available relative to animal demand. Unlike finishing systems where the primary goal is maximizing individual animal gain, cow–calf operations must balance individual animal performance with production per unit area to achieve economic viability [14]. This balance is mediated through FA, which directly influences forage intake, the primary determinant of animal performance in grazing systems [15]. Research consistently demonstrates that the relationship between FA and pasture intake is positive but curvilinear, with intake increasing as allowance increases but with diminishing returns at higher allowances [14,16,17].

In this study, we hypothesize that FA, determined from satellite-derived standing FM and stocking rate based on cow herd size, is the key factor influencing dry matter intake (DMI) in grazing systems, as it establishes the link between the animal component and the pasture component within these systems. Cow DMI directly influences milk yield and, consequently, calf weight gain. Changes in calf growth affect WW, which, together with the number of calves produced, determines system-level productivity, profitability, and environmental outcomes. Therefore, this study aimed to develop a DST framework, CattleSat, that integrates satellite-derived standing FM with ruminant nutrition models to determine the optimal cow herd size that maximizes WW production while minimizing environmental impact and optimizing profitability based on historical patterns of forage availability.

## 2. Materials and Methods

Because no human or animal subjects were used, this analysis did not require approval by Institutional Animal Care and Use Committee or Institutional Review Board.

Section 2.1 describes the development of the model framework, including its variables and user-adjustable parameters. Section 2.2 presents a case study simulation demonstrating how the model can be applied.

### 2.1. Overall Description of the Model

The CattleSat model is a hybrid mathematical model that integrates several submodels to assess various aspects of a cow–calf grazing system (Table 1) by combining satellite-derived standing FM from machine learning (ML) models and mechanistic ruminant nutrition models (Ruminant Nutrition System, RNS; [18]) with empirical equations to estimate herd management, greenhouse gas (GHG) emissions, and profitability. Equations are described in the main text and in Appendix A.

**Figure 1:**
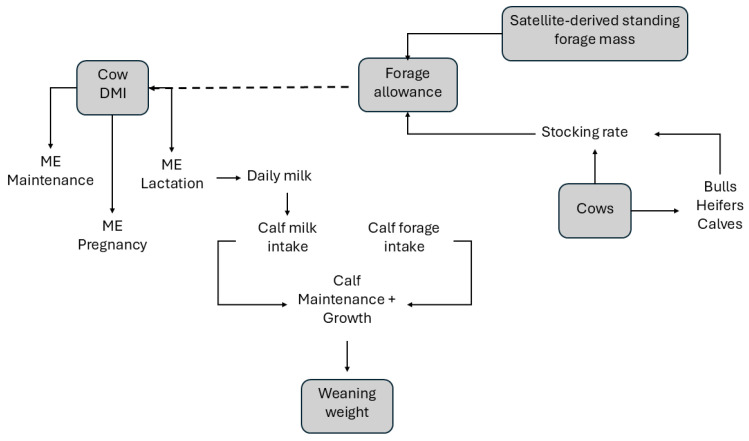
Flowchart of main variables and their interrelationships to determine the optimal cow herd size to maximize total weaning weight based on forage availability.

The core structure of the model, representing the interaction between cow herd size and forage availability to maximize the total WW, is illustrated in Figure 1. Cow herd is the central variable that influences the number of bulls, heifers, and calves, and, ultimately, the stocking rate. Standing dry FM, determined using satellite-derived models, and stocking rate directly impact FA, our model’s main driver of animal forage intake. Calf live weight gain is a key factor in the model, influenced by daily milk yield (MY), driven by the metabolizable energy available for lactation [19]. The ultimate goal is to maximize WW, which depends on the calf forage intake and the energy available for milk production.

#### 2.1.1. Forage and Grazing Submodel

***Satellite-derived standing dry forage mass.*** In grazing nutrition models, the first limiting factor is forecasting the actual availability of pasture forage and its correlation with DMI. In our model, we assumed that the monthly dynamic of FM is obtained using previously published models and free or commercial platform providers [11,20,21] in a timeframe of at least the past 5 years.

Forage Allowance. Forage allowance is estimated monthly according to Sollenberger et al. [13] by dividing total dry FM by total animal live weight (Equation (1))

FA_m_ = FM_m_ × area/(n Cow_m_ × BW cow_m_ + n Bull_m_ × BW bull_m_ + n 1st Heifer_m_ × BW 1st Heifer_m_ + n Heifer_m_ × BW heifer_m_ + n Calf_m_ × BW calf_m_) × 1000, (1)

where m = month 1 to 12; FA = instantaneous DM-based forage allowance (kg DM/kg BW) in month m, FM = satellite-derived standing dry forage mass (g/m^2^) in the month m, area = m^2^, n = head of animals in each category in month m, BW = body weight (kg) of animals in each category in month m, and 1st heifer = first-calf heifer.

Hay Supplementation. Hay is assumed to be provided for bred cows (the sum of mature cows and first-calf heifers) and replacement heifers during the months of November, December, January, and February and user-adjustable to the proportion provided to animals (0–100% DMI).

Concentrate and Mineral Supplementation. This model assumed an optional daily concentrate supplementation for first-calf heifers and yearling heifers and an optional daily creep-feeding supplementation for 60 days for calves. Mineral supplementation was assumed to be 35, 30, 20, 40, and 10 g per day for cows, first-calf heifers, yearling heifers, bulls, and calves, respectively.

#### 2.1.2. Management Submodel

Herd Size and Stocking. In this conceptual model, the cow herd (number of cows) is the primary driver of total herd size, including bred cows, bulls, replacement heifers, first-calf heifers, and calves. The model begins with the number of cows specified by the user and simulates how changes in herd size influence WW, profitability, and carbon emissions. These outcomes are estimated using historical satellite-derived FM data from the pasture.

The number of bulls is calculated by multiplying the number of cows by the assumed cow-per-bull ratio (default value = 30). Replacement heifers are estimated as a proportion of the cow herd based on the replacement rate (default value = 30%), and first-calf heifers are assumed to represent half of the replacement heifers. Calf numbers are estimated by multiplying the combined total of cows and first-calf heifers by the birth rate (default value = 83%) and the survival rate (1 − death rate, default value for death rate = 6%/year).

#### 2.1.3. Animal Nutrient Requirements and Dry Matter Intake Submodel

The RNS was used to estimate animal nutrient requirements, DMI, MY, and WW [18]. The main equations used in the model are described in the main text and Appendix A.

Maintenance. The net energy requirement for maintenance (NEm) for all animals was assumed to be the basal metabolism adjusted for the previous plane of nutrition, sex, previous temperature, lactation, body condition score, and physical activity (Equations (A1)–(A5), Appendix A). The ME requirement for maintenance was calculated by dividing ME by partial efficiency of ME use for maintenance (Equation (A6), Appendix A; [18]).

Pregnancy. The daily net energy requirements for pregnancy (NEpr) were estimated based on expected calf birth weight (CBW), days of gestation, and a standard CBW value assumed to be 41 for beef calves (Equations (A7) and (A8), Appendix A) according to Tedeschi and Fox [18]. The ME requirement for pregnancy was calculated assuming a partial efficiency of ME use for pregnancy of 13% [18].

Lactation and milk production. The average daily MY was calculated based on ME available for lactation (MElr) after subtracting the ME requirement for maintenance and pregnancy from the metabolizable energy intake (MEI) as shown in Equations (A9)–(A12) (Appendix A). The net energy requirement for lactation (NEl) was calculated assuming a partial efficiency of ME use for lactation of 64.4% [18]. Milk composition is used to compute net energy in milk, which drives the energy requirement for lactation [19]. Then, the MY were estimated based on NEl, milk fat, lactose, and true protein content [18], as shown in Equation (A9) (Appendix A).

Growth. Body weight gain of heifers and calves was estimated according to Tedeschi and Fox [18]. The daily dietary net energy available for growth (NEg) was estimated based on the diet ME according to NASEM [4]. The partial efficiency of ME use for growth was estimated as NEg/ME [18]. A fixed value of 5.29 Mcal of ME/kg of milk (dry matter (DM) basis) is assumed in computing the intake of ME by the calf [19]. The NEg was then used to predict daily BW gain (Equations (A13) and (A17), Appendix A). The WW is assumed to be the calf BW at 240 days of age.

Intake. In this model, the DMI of cows, bulls, and heifers is estimated based on forage intake adjusted for temperature, mud depth, and feed additives, according to Tedeschi and Fox [18] (Equations (2)–(8)), and adjusted to FA (Equation (9)).

DMI = (DMI_for_ × Adj_MF_ × Adj_TF_ × Adj_BF_ × Adj_AD_) + Adj_FA_DMI_ (2)

where DMI = dry matter intake (kg/d), DMI_for_ = potential forage DMI, Adj_MF_ = adjustment factor of predicted DMI for mud (dimensionless), Adj_TF_ = adjustment factor of predicted DMI for temperature (dimensionless), Adj_BF_ = adjustment factor of predicted DMI for breed (dimensionless), Adj_AD_ = adjustment factor of predicted DMI for feed additives (dimensionless), and Adj_FA_DMI_ = adjustment factor of predicted DMI for FA (kg/d).

Forage intake (DMI_for_) uses empirical equations described in Tedeschi and Fox [18,19] based on BW and forage digestibility (Equations (3)–(5)). Generally, pregnant and dry beef cows consume 1.8 to 2% of their BW as daily DM, while lactating beef cows consume approximately 2.3 to 2.5% of their BW as daily DM [18].

DMI_for-a_ = (4.56 + 0.0053 × BW − 0.00002 × BW^2^ − 0.05531 × D + 0.00032 × BW × D)/OM_for_ (3)

DMI_for-b_ = (12.5 − 0.0299 × BW + 0.00002 × BW^2^ − 0.05531 × D + 0.00032 × BW × D)/OM_for_ (4)

DMI_for-c_ = (27.9 − 0.0902 × BW + 0.00009 × BW^2^ − 0.05531 × D + 0.00032 × BW × D)/OM_for_ (5)

where DMI_for_ = forage dry matter intake (kg/d), a = for growing cattle, heifers; b = for dry cows, bulls; c = for lactating cows; BW = body weight (kg); D = forage digestibility (%), DMI = dry matter intake (kg/d), and OM_for_ = organic matter content of forage (g/100 g).

Adj_MF_ = 1 − 0.01 × Mud depth (6)

Adj_TF_ = a + b × (2 × e × [Ln[exp((CETI + d/2)/e) + exp(c/e)] − Ln[exp((c + d/2)/e) + exp(CETI/e)]] + d)/(2 × d) (7)

a = 0.9, b = 0.1, c = 24.7, d = −0.00145, e = −0.96 for CETI ≥ 29 °C and LNT ≥ 20 °C.

a = 0.65, b = 0.25, c = 35.5, d = 0.33, e = −1.21 for CETI ≥ 29 °C and LNT < 20 °C.

a = 0.9, b = 0.1, c = 24.7, d = −0.00145, e = −0.96 for 20 °C ≤ CETI < 29 °C.

a = 1, b = 0.03, c = 14.5, d = −0.0019, e = −0.9 for 10 °C ≤ CETI < 20 °C.

a = 1.03, b = 0.02, c = 4.5, d = −0.0019, e = −0.9 for 0 °C ≤ CETI < 10 °C.

a = 1.05, b = 0.02, c = −5.5, d = −9.7 × 10^−5^, e = −0.85 for −10 °C ≤ CETI < 0 °C.

a = 1.07, b = 0.09, c = −14.4, d = 0.0046, e = −1.04 for CETI < −10 °C.

CETI = 27.88 − 0.456 × T_c_ + 0.010754 × T_c_^2^ − 0.4905 × RH_c_ + 0.00088 × RH_c_^2^ + 1.1507 × WS − 0.126447 × WS^2^ + 0.019876 × T_c_ × RH_c_ − 0.046313 × T_c_ × WS + 0.4167 × HRS (8)

Adj_BF_ = 1.08 For Holstein

=1.04 For Holstein × beef breeds

=1 For Other breeds

Adj_AD_ = 0.9118 For monensin, no implants

=0.94 For none, lasalocid, laidlomycin, no implants

where Adj_MF_ = adjustment factor of predicted DMI for mud (dimensionless), mud depth = cm, Adj_TF_ = adjustment factor of predicted DMI for temperature (dimensionless), CETI = current effective temperature index (°C), T_c_ = current temperature (°C), RH_c_ = current relative humidity (%), WS = wind speed (km/h), HRS = hours exposed to direct sunlight (h), LNT = least night temperature (°C), Adj_BF_ = adjustment factor of predicted DMI for breed (dimensionless), and Adj_AD_ = adjustment factor of predicted DMI for feed additives (dimensionless).

To adjust the intake according to FA, we relied on the long-term grazing study of Rouquette and collaborators [17], who demonstrated that the relationship between FA and average daily gain (ADG) of suckling calves and lactating cows under continuously stocked bermudagrass systems was best represented by a two-phase linear response. In their study, ADG increased as FA increased up to an inflection point, beyond which additional FA produced little or no additional improvement in animal performance. Because ADG is fundamentally driven by nutrient and dry matter intake, we adopted the same biological concept in this study to represent the effect of FA on DMI. Thus, the broken-line function (linear, plateau; [17]) was used to describe a biologically plausible response in which DMI increases with forage availability until reaching a plateau (maximum FA, FA_max_) associated with intake limitation or animal physiological capacity, above which there is no impact on DMI, and this is described as follows (Equation (9)).

When FA < FA_max_:

Adj_FA_DMI_ = factor × (FA − FA_min_) (9)

where Adj_FA_DMI_ is the adjustment of DMI related to FA (kg/d), factor is the slope representing the change in DMI for each change in FA (kg DMI/FA), FA_min_ is the minimum FA of the grazing ecosystem, and FA_max_ is the maximum (plateau) FA of the grazing ecosystem, beyond which additional FA produced no additional improvement in DMI. The factors FA_min_ and FA_max_ depend on type of pasture, region, grazing management, and other interventions (e.g., nitrogen fertilization). These parameters are assumed to be calibrated according to specific pasture and grazing conditions.

The forage intake of nursing calves is computed in our model based on the model developed by Tedeschi and Fox [19]. The MEI was estimated as the product of DMI and metabolizable energy of the diet/forage (ME_diet_, Mcal/kg DM).

#### 2.1.4. Emissions Submodel

Environmental impact is assessed using estimates of GHG emissions from a life cycle assessment. Systems boundaries are limited to cradle-to-farm, with a 365-day timeframe. Accounted GHG emissions are calculated as the sum of CH_4_ emissions from enteric fermentation and feces, nitrous oxide (N_2_O) from animal excreta (manure and urine), and fossil carbon dioxide (CO_2_) from animal feed and fertilizer production, manufacturing, and transportation. Other emissions related to farm operations, power generation, buildings and machinery, veterinary and pesticide products, and emissions beyond the farm gate (such as transportation to slaughterhouses and carcass processing) are not included in the model. Soil organic carbon is assumed to be at equilibrium.

Enteric CH_4_ emissions, CH_4_ emissions from feces, and direct and indirect N_2_O emissions from excreta and N-based fertilizer were estimated using the Tier 2 IPCC methodology [22]. Enteric CH_4_ emissions are modeled using Equation (10.21) and Table 10.12 from Tier 2 refinement methods in Chapter 10 of IPCC [22]. The CH_4_ emissions from feces are determined based on total fecal production from DMI and diet digestibility using Equations (10.23) and (10.24) from Chapter 10 of IPCC [22]. Nitrogen excreted through feces and urine is estimated based on the nitrogen retention fraction from Table 10.20 in Chapter 10 of the IPCC [22] and nitrogen intake. The nitrogen intake is obtained by multiplying DMI by diet crude protein and dividing by a factor of 6.25. Direct N_2_O emissions from grazing cattle are estimated using the default emission factor (EF_3PRP_) recommended in Chapter 10 of IPCC (Table 10.21 of IPCC [22]). Indirect N_2_O emissions are estimated based on emission factors (EF) recommended in Chapter 11 (Table 11.3 of IPCC [22]). The EF from feed are obtained from sources identified and cited in the literature ([23,24]; Supplementary Data S1 Table S1). All emissions are converted to their 100-year global warming potential in CO_2_e, which are 27 and 273 for CH_4_ and N_2_O, respectively [25]. Emissions are allocated as a function of BW production (kg CO_2_e/kg BW) estimated by the difference between final and initial total BW of weaned calves and heifers.

#### 2.1.5. Economics Submodel

Profitability. The total annual cost for each simulated target WW was estimated by summing the variable costs of hay supplementation, concentrate supplementation, salt and minerals, veterinary expenses, marketing expenses, fuel, lube, repairs, and labor, together with fixed pasture costs. These costs were based on the budgets of Texas A&M AgriLife Extension Agricultural Economics [26] for District 8 cow–calf native pasture operations. The revenue was determined by multiplying the total weaned calf weight by calf sale price, while net income was estimated as the difference between revenue and total cost. Assumptions used to simulate financial outputs were based on actual hay and calf prices ([27] and other costs from Texas A&M AgriLife Extension Service enterprise budgets (Supplementary Data S1 Table S2).

### 2.2. Case Study

Data acquired from Texas A&M University McGregor Research Center, a pasture-based cow–calf operation located in McLennan County, Texas, was used as a testbed for the model. As the pasture herbaceous matter was the focus of this evaluation, McGregor Research Center was selected based on data availability and the lack of tree canopy. The region is characterized by subtropical climatic conditions, with average temperatures ranging from 2 to 24 °C and year-round rainfall that is highly variable. The operation spans 2578 ha, of which 1461 ha are native grass pastures, 479 ha are improved pastures, 390 ha are used for row crops, and 248 ha are used for hay production. Approximately 40% of the pasture area has mild-to-high weed infestation. Slidell silty clay, Sanger clay, McLennan clay loam, and Crawford silty clay are the predominant soils [28]. We assumed continuous stocking and no pasture fertilization for this simulation. Herd inventory from 2017 to 2022 is depicted in Table 2. Calving generally occurs from February to April, and calves are weaned, on average, in October or November.

Pasture forage data from 2017 to 2023 were retrieved from satellite imagery. First, pastures from McGregor Research Center were geo-referenced to the WGS84 UTM map projection using an open-source image processing package (QGIS Desktop version 3.22.9, QGIS Organization, Berne, Switzerland). The geo-referenced pasture maps were then provided to SigFarm Intelligence LLC (College Station, TX, USA), which generated monthly predictions of standing pasture biomass using algorithms based on Fernandes et al. [20]. The commercial platform uses Sentinel-2 satellite imagery, and its FM prediction algorithm is based on the Extreme Gradient Boosting (XGBoost) model, which achieved an R^2^ of 0.6051 and a root mean square error (RMSE) of 48.15 g/m^2^. To further refine forage estimates, we incorporated data from the Rangeland Analysis Platform (RAP; [21]). The RAP data were used to adjust FM estimates based on the proportion of herbaceous vegetation cover (10-meter resolution) and pasture growth measured as 16-day aboveground biomass production (Table S3, Supplementary Data). Maps derived from satellite imagery showed spatiotemporal variations in available FM across all paddocks, consistent with expected changes in pasture vegetation driven by forage development, management strategies, and weather conditions. Pasture digestibility (DM basis) was assumed to be 56% from April to June, 48% from July to August, and 43% DM from September to March [7]. Pasture DE and ME was assumed to be 2.50 and 2.05 Mcal/kg DM from April to June, 2.12 and 1.74 Mcal/kg from July to August, and 1.89 and 1.55 Mcal/kg DM from September to March [7], respectively.

In this simulation, the coefficients used to adjust DMI for FA were 0.85, 3.0, and 4.5 for the factor, FA_min_, and FA_max_, respectively (see Equation (2)). The factor coefficient was derived from previous research on native pastures [14,16], which represent the majority of the pastures at McGregor. The FA_min_ and FA_max_ values were determined based on a calibration of our model (Table S4, Supplementary Data) using the on-farm observational data from 2017 to 2022 provided in Table 2, by first defining the range of monthly observed FA (1.47 to 4.55 kg DM/kg BW) and subsequently iteratively adjusting parameter (FA_min_ and FA_max_) values within this range until model-predicted WW aligned with observed WW from the production system.

The default values (user-adjustable values) used in the case study simulation are depicted in Table 3. We included fixed default management inputs to represent a typical cow–calf production system in the study region and based on the information provided from the Texas A&M University McGregor Research Center. Additional parameters and their default values related to weather data, the emissions submodel, and the economics submodel are provided in the Supplementary Data S1 (Tables S1, S2, and S5).

The ME of hay was assumed to be 1.70 Mcal/kg DM. This model assumed a daily concentrate supplementation (as dried distillers’ grain cubes, DE of 3.8 Mcal/kg DM) at a rate of 2 kg/animal for 161 days for first-calf heifers and yearling heifers and no supplementation for calves.

We simulated the cow herd beginning with 650 cows, increasing by increments of 20%, 40%, 60%, 80%, and 100% (780, 910, 1040, 1170, 1300 cows, respectively).

Sensitivity Analysis. To assess the variability and key drivers affecting productivity and economic performance, a Monte Carlo simulation was conducted using the @Risk^®^ software v8.2 (Palisade Corp., Ithaca, NY, USA). Each scenario was simulated over 1000 iterations. Stochastic inputs included cow BW, pregnancy rate, monthly FM, maximum and minimum FA, and market prices for calves and hay, assuming normal distributions (Table S6, Supplementary Data). Output variables were WW, net return, and net return per cow. The sensitivity analysis was performed using regression coefficients derived from Latin Hypercube sampling to identify the relative influence of each input factor on the outputs. This approach allowed for quantification of the robustness of model predictions and identification of priority variables that most affect productivity and profitability in cow–calf systems.

## 3. Results

**Figure 2:**
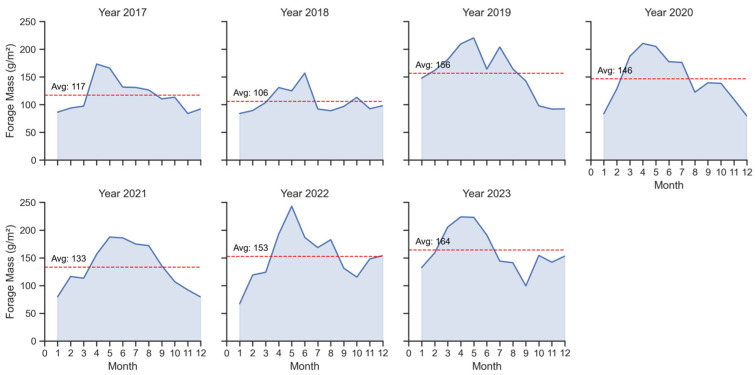
Monthly satellite-derived standing dry forage mass from 2017 to 2023 at Texas A&M University McGregor Research Center (McGregor, TX, USA). Red dashed lines represent the annual average.

Satellite-derived dry FM exhibited clear seasonal and interannual variability across the 2017–2023 period (Figure 2). Forage mass followed a consistent seasonal pattern, with peak values occurring during spring (between March and May) and declining through late summer and winter. However, substantial variation among years was observed, particularly in the magnitude and timing of peak biomass. Annual averages (Figure 2) indicate that some years exhibited markedly higher forage availability, reflecting differences in climatic conditions and pasture growth dynamics.

**Figure 3:**
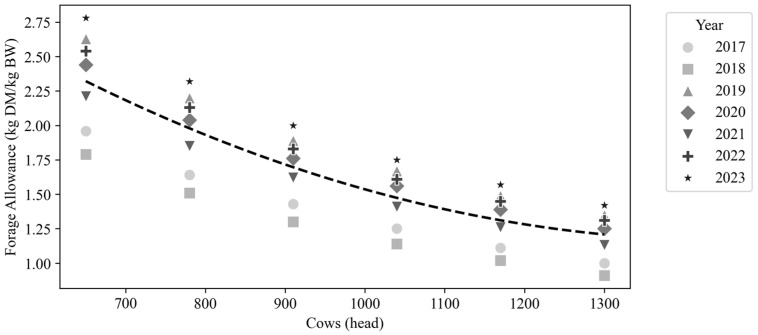
Effect of cow herd size (head of mature cows) on annual average dry forage allowance (kg forage dry mass/kg body weight) based on satellite-derived measurement of available forage from 2017 to 2023.

Our model consistently shows a sharp decline in FA as herd size increases (Figure 3) due to greater grazing pressure relative to available forage. This reduction was nonlinear, with the most pronounced decreases occurring at higher stocking densities. This finding highlights the sensitivity of FA to stocking decisions and reinforces its role as a key intermediary variable linking pasture supply and animal demand.

**Figure 4:**
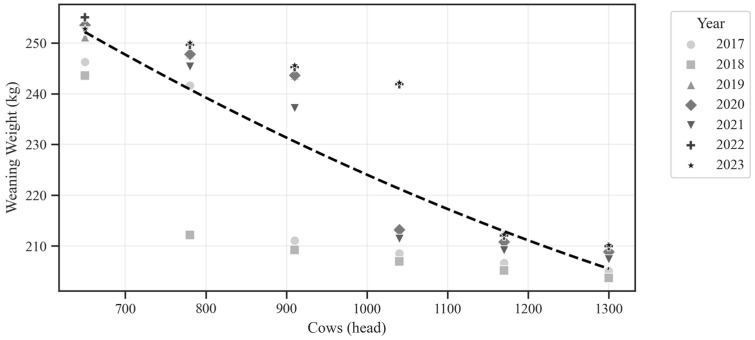
Effect of cow herd size (head of mature cows) on the expected weaning weight based on satellite-derived measurement of available forage from 2017 to 2023.

**Figure 5:**
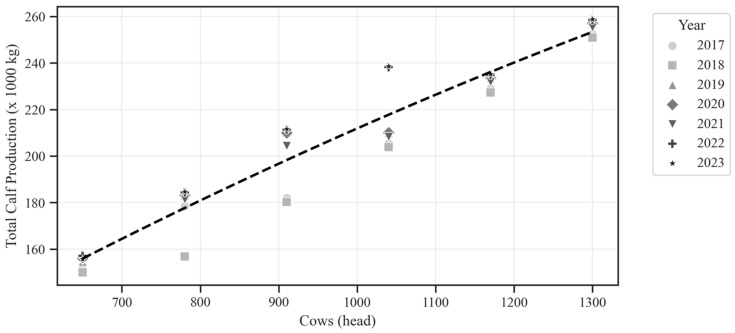
Effect of cow herd size (head of mature cows) on the total calf production (×1000 kg) from 2017 to 2023.

Consequently, the decrease in FA due to greater herd size directly results in decreased individual animal performance, expressed as WW (Figure 4), reflecting the reduced forage intake per animal under lower FA conditions. This relationship illustrates the trade-off between stocking rate and individual animal productivity, where larger herd sizes compromise per-animal performance due to increased competition for limited forage resources. In contrast, total calf production exhibited a curvilinear response to herd size (Figure 5). In our simulation, total calf production (product of number of calves multiplied by WW and calf market price) increased with herd size from 650 to 1300 cows.

**Figure 6:**
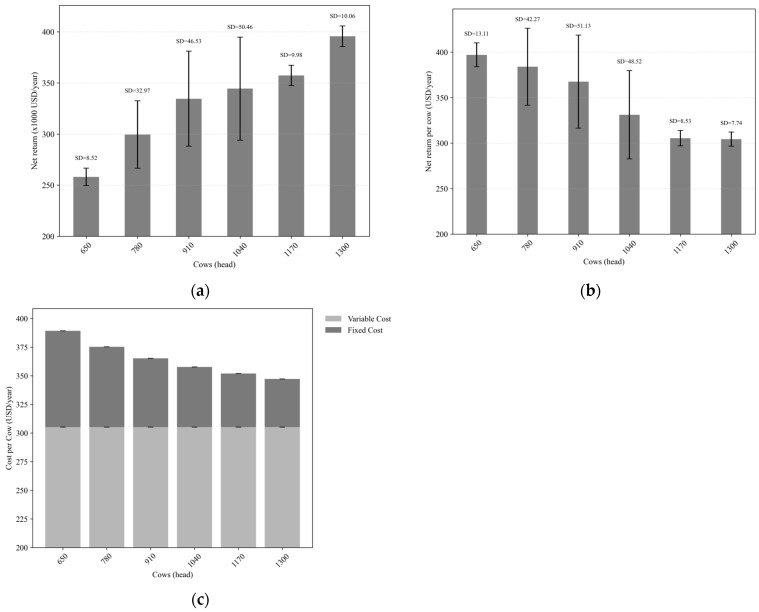
Effect of cow herd size (head of mature cows) on (**a**) net return (×1000 USD/year), (**b**) net return per cow (USD/cow/year), and (**c**) cost per cow (USD/cow/year). Bars in (**a**–**c**) represent the standard deviation (SD) across the years 2017 to 2023.

Economic outcomes followed similar nonlinear patterns (Figure 6). Total net return (Figure 6a) increased with herd size up to 1300 cows, but net return per cow (Figure 6b) decreased progressively as herd size increased, indicating reduced individual efficiency at higher stocking rates. Cost per cow (Figure 6c) decreased as herd size increased, reflecting the dilution of fixed costs across a larger number of animals. The variability across years, represented by standard deviations, highlights the influence of interannual forage variability and market conditions on profitability.

**Figure 7:**
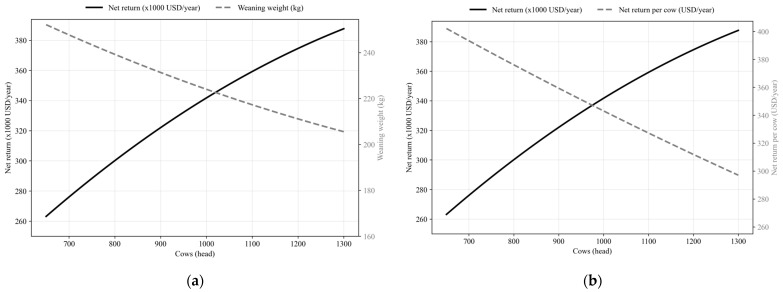
The relation between net return and weaning weight (**a**) and net return per cow (**b**) according to the cow herd size (head of mature cows).

The relationship between productivity and profitability is further illustrated in Figure 7. The relationship between net return and WW (Figure 7a) and between net return per cow and WW (Figure 7b) revealed a crossing point across herd sizes. The crossing curves converged at approximately 900 to 1000 cows, indicating a transitional stocking level at which system-level profitability and individual animal efficiency were balanced. Below this range, increases in herd size resulted in simultaneous gains in both total net return and net return per cow, driven by improved utilization of available forage. Above this threshold, total net return continued to increase or stabilize, while net return per cow declined, reflecting reduced individual performance due to decreasing FA. This crossing point, therefore, may represent a critical management point where the marginal benefits of increasing herd size begin to shift from individual efficiency toward system-level output.

**Figure 8:**
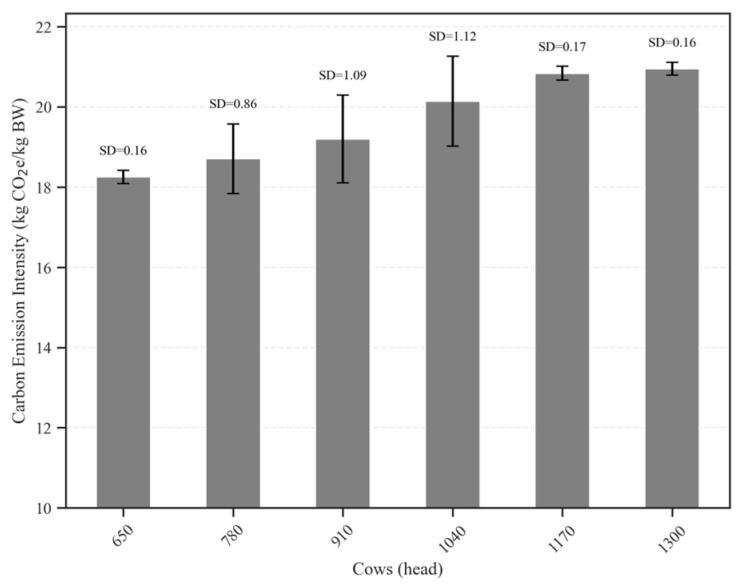
Effect of cow herd size (head of mature cows) on carbon emission intensity per body weight (kg CO_2_e/kg BW). CO_2_e = CO_2_ equivalent, BW = body weight. Bars represent the standard deviation (SD) across the years 2017 to 2023.

Carbon emission intensity increased with herd size (Figure 8), indicating reduced environmental efficiency at higher stocking rates. As herd size increased and WW declined, emissions per unit of output (kg CO_2_e/kg BW) increased.

**Figure 9:**
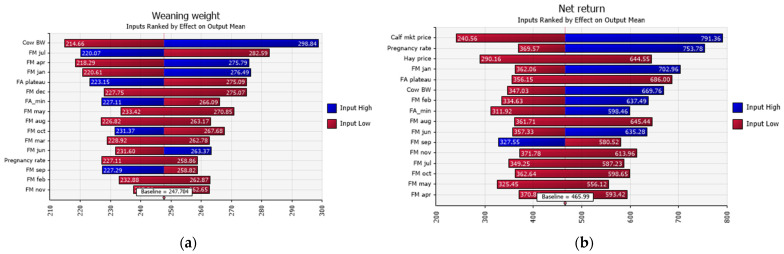
Effect of the most influential input variables on (**a**) weaning weight and (**b**) net return (×1000 USD/year) derived from Monte Carlo simulations (1000 iterations) using @Risk^®^. BW = body weight, FM = pasture forage mass, FA = forage allowance.

The sensitivity analysis identified the most influential input variables affecting system outputs (Figure 9). Weaning weight (Figure 9a) was primarily driven by cow BW followed by forage-related variables, including FM and FA parameters, confirming their central role in determining intake and growth (Table S7, Supplementary Data). Net return (Figure 9b) was strongly influenced by both biological and economic variables, particularly calf price, hay price, and FM. Overall, our model indicates that forage dynamics and market conditions are the primary drivers of variability in productivity and profitability within the system.

## 4. Discussion

The present study highlights the central role of forage availability and stocking decisions in shaping the biological, economic, and environmental outcomes of cow–calf systems. The pronounced seasonal and interannual variability in FM observed in Figure 2 is consistent with previous studies demonstrating that pasture productivity in the southern United States is strongly influenced by climatic conditions and management practices. Variations in precipitation and temperature directly affect forage accumulation, nutritive value, and grazing capacity, thereby influencing animal performance and overall system efficiency [29,30]. Recent assessments of pasture and rangeland conditions in Texas further underscore these challenges, indicating a concerning shift toward poorer pasture quality. Over the past decade, a growing proportion of the state’s grazing lands have been classified as poor or very poor, with approximately 7 million hectares transitioning from good or fair condition since 2016 [27]. Although interannual climatic variability contributes to these trends, prolonged overgrazing and excessive stocking rates are recognized as major drivers of pasture degradation and the proliferation of invasive plant species [30]. In this context, the present model incorporates satellite-derived estimates of FM to explicitly account for spatiotemporal variability in forage supply and to evaluate its interaction with stocking rate on system performance.

In our model, FA is the central mechanism linking pasture dynamics to animal performance, economic returns, and environmental outcomes in cow–calf grazing systems. In this context, FA acts as the primary regulator of DMI [14,16], in which the seasonal and interannual variability in FM directly influenced FA and, consequently, intake and animal performance. Years with greater forage availability resulted in higher FA values, allowing animals to achieve near-maximal intake and performance. In contrast, years with reduced FM constrained FA and limited intake, reinforcing the importance of accurately capturing pasture dynamics when modeling grazing systems. This variability highlights the advantage of integrating satellite-derived data as it enables continuous monitoring of forage conditions that are otherwise difficult to quantify [11].

The inverse relationship observed between herd size and FA (Figure 3) aligns with established principles of grazing management. Increasing stocking rates intensify grazing pressure, reducing available forage per animal and constraining intake. This mechanism explains the decline in individual animal performance (Figure 4), particularly WW, as herd size increases. Similar trade-offs have been widely documented, where increased stocking rates reduce individual gains but may increase production per unit area up to a threshold [17].

Our results were consistent with those of Rouquette et al. [17], despite the differences in pasture ecosystem. Rouquette et al. [17] showed the significant impact of FA on the performance of both beef cows and calves in bermudagrass grazing systems. Their 11-year grazing study found that adequate FA levels, specifically, 1.2 to 1.5 kg DM/kg BW in spring and 1.5 to 2.0 kg DM/kg BW in summer, supported optimal calf gains. Performance was limited at low FA, while excessively high FA did not proportionally improve gains, indicating diminishing returns. Rouquette et al. [17] also demonstrated a two-phase response in calf ADG to FA, with diminishing returns at higher allowances, a trend that was indirectly reflected in our model’s sensitivity to interannual forage variation.

The curvilinear response of total calf production (Figure 5) reflects the balance between individual animal performance and production per unit area. At low stocking rates, FA exceeds the threshold at which intake is no longer limiting, resulting in high individual performance but underutilization of forage resources. As herd size increases, FA declines toward an optimal range, increasing total production. However, further increases in herd size reduce FA below critical thresholds, limiting intake and reducing individual performance, which offsets gains from additional animals. This response is consistent with the classical grazing theory but is explicitly generated in our model through the FA–DMI relationship embedded in the model.

Importantly, increasing cow size and associated maintenance requirements further complicate this relationship. Larger cows require substantially more energy intake and forage consumption, which can reduce system efficiency if not matched with adequate forage resources [29]. Therefore, the optimal stocking rate is dynamic and must account for both animal requirements and forage supply. The decline in WW with increasing herd size also reflects underlying nutritional constraints and maternal effects. Milk production is a key determinant of pre-weaning growth; however, its relationship with overall system efficiency is complex. While greater MY is generally associated with increased WW, it may also increase metabolic demands on the cow and affect reproductive efficiency under limited forage conditions [31,32]. This suggests that selecting for higher milk production without considering forage availability may not improve whole system productivity.

Economic outcomes observed in this study (Figure 6) further emphasize the importance of balancing stocking rate and resource availability. In our simulation, total net return increases with herd size up to 1300 cows, while net return per cow steadily declines, indicating that individual efficiency is more sensitive to FA constraints than total system output. The relationship between productivity and profitability is further clarified by the crossing curves observed in Figure 7. This crossing point, occurring at approximately 900 to 1000 cows, may represent a critical transition point in system behavior. Mechanistically, this point could correspond to FA declining from values near or above FA_max_ (≈4.5 kg DM/kg BW) into the intermediate range between FA_max_ and FA_min_ (≈3.0 kg DM/kg BW), as assumed in Equation (3). At lower herd sizes, FA is sufficiently high that DMI is not constrained, allowing both total net return and net return per cow to increase simultaneously. As herd size approaches the crossing point, FA enters a region where intake begins to be moderately restricted, resulting in a balance between system-level profitability and individual animal efficiency. Beyond this point, FA declines below FA_min_, triggering a stronger reduction in DMI, which leads to decreased WW and a decline in net return per cow, even if total net return remains stable or increases. Therefore, the crossing point in Figure 7 could be indicative of a transition point from a non-limiting to a limiting intake regime, governed by the FA–DMI function.

The increase in carbon emission intensity with higher stocking rates reflects reduced production efficiency. As animal performance declines, emissions per unit of output increase, highlighting a key trade-off between intensification and environmental sustainability [33,34,35]. However, livestock sustainability should be interpreted within a broader systems perspective that considers not only GHG emissions, but also food security, nutrient cycling, ecosystem services, rural livelihoods, and the use of lands unsuitable for crop production [36]. Previous studies have emphasized that livestock systems contribute to complex socioecological functions and that evaluating sustainability exclusively using emission metrics may lead to overly reductionist interpretations of ruminant production systems [36,37]. Furthermore, climate change discussions related to livestock production should also consider the role of adaptive management strategies, resilience of grazing systems, and the balance between productivity and environmental stewardship. In this context, improvements in forage management, such as rotational grazing, stockpiling, and the integration of cool season annuals, have been shown to increase forage use efficiency and reduce reliance on external inputs, thereby improving both economic and environmental outcomes [30,38]. These strategies are critical for achieving sustainable intensification in grazing systems.

The sensitivity analysis underscores the dominant role of forage-related variables in determining system performance. Forage mass and FA were important drivers of WW and economic returns, highlighting the strong influence of pasture dynamics on cow–calf productivity. In addition, economic variables such as calf prices significantly influenced profitability, reflecting the exposure of producers to market risks. This aligns with findings from Rourke et al. [39] who identified economic and marketing risks as the areas with the largest knowledge gaps among cow–calf producers. Nonetheless, the strong influence of FA-related parameters further emphasizes the importance of accurately parameterizing the intake response function, as small changes in FA thresholds can substantially alter system outcomes.

Despite the strengths of the CattleSat framework, future studies and improvements should be incorporated to overcome some of its current limitations. First, the model relies on satellite-derived estimates of FM, which, although validated in previous studies, remain subject to uncertainties related to sensor resolution, cloud cover, and the accuracy of ML algorithms used to predict biomass. Additionally, the model assumes uniform forage quality within each time period and does not explicitly account for spatial heterogeneity in nutritive value or selective grazing behavior, which may influence intake and animal performance under field conditions. Future research should then focus on improving the representation of pasture heterogeneity by integrating higher-resolution remote sensing data and incorporating dynamic forage quality predictions.

Because this study primarily focused on model development and framework integration, the McGregor case study should be interpreted as a demonstration of model applicability rather than a comprehensive independent validation. Parameters related to the FA–DMI relationship, such as FA_min_ and FA_max_, were calibrated using the available dataset to represent the grazing conditions evaluated in this study. Therefore, additional validation using independent datasets, production environments, pasture types, and grazing systems is necessary before broader application of the framework.

In our model, the DMI response to FA is represented using a simplified broken line function (Equation (3)) defined by fixed parameters (FA_min_, FA_max_, and slope) that depend on pasture type, botanical composition, or grazing management. Consequently, future studies should focus on the refinement of the FA–DMI relationship using region-specific calibration datasets to enhance model accuracy and applicability across diverse grazing systems. Moreover, coupling the model with stochastic market simulations and risk analysis frameworks would improve its utility as a DST under uncertain economic and climatic conditions.

## 5. Conclusions

From a management perspective, our model framework demonstrates the potential utility of integrating pasture monitoring with adaptive, data-driven stocking strategies under variable forage conditions. Fixed stocking rates are unlikely to be optimal under variable forage conditions, particularly in environments characterized by high climatic variability. Instead, flexible approaches that adjust herd size or supplementation based on forage availability can improve system resilience. Additionally, improving forage systems through diversification and intensification can enhance carrying capacity while maintaining or improving animal performance and resource use efficiency.

Overall, this study illustrates how integrating satellite-derived pasture information with nutrition, environmental, and economic models can support the evaluation of trade-offs among productivity, profitability, and environmental outcomes in grazing systems. However, because this study focused primarily on model development and a demonstration of applicability, additional calibration and external validation across independent farms, pasture types, management systems, and climatic regions are required before broader application of the framework under commercial conditions.

## Figures and Tables

**Table 1:** Built-in submodels of the cow–calf nutrition model (CattleSat), their main model type, inputs (user-adjustable parameters), and main outputs.

Submodel	Model Type	Inputs	Outputs
Forage and Grazing	Satellite-based machine learning	Multipolygon, area	Dry forage mass
Management	Empirical	Initial cow herd, cow-per-bull ratio, birth rate, death rate, replacement rates, BW	Cows, bulls, heifers, calves, weaned calves, stocking BW
Animal Nutrient Requirements and Dry Matter Intake	Mechanistic	Weather data, animal features (breed, sex, BW), diet chemical composition	DMI, milk yield, calf BW gain, WW
Emissions	Empirical	CP diet, fecal OM excretion, emission factors	Carbon emissions (CO_2_e), emission intensity (CO_2_e/kg weaned calves)
Economics	Empirical	Feed DMI, feed price, veterinary and overhead costs, calf price	Cost, net income

BW, body weight;CO_2_e, carbon dioxide equivalent; CP, crude protein; DMI, dry matter intake; OM, organic matter; WW, weaning weight.

**Table 2:** Characteristics of the Texas A&M University McGregor Research Center herd from 2017 to 2022.

	2017	2018	2019	2020	2021	2022
Cows, n	794	838	889	965	1037	1006
1st Calf Heifers, n	178	100	118	97	171	104
Replacement Heifers, n	314	367	100	252	205	250
Calves Born, n	775	846	853	965	1043	762
Weaned Calves, n	755	810	829	946	991	709
Timing of Calving	Spring	Spring	Spring	Spring	Spring	Spring
Weaning Age, days	228	191	219	226	230	181
Weaning Weight ^1^, kg	249.6	223.3	221.2	215.9	230.9	227.7
Forage Allowance ^2^, kg/kg BW	2.24	2.07	3.55	2.76	2.66	2.33

^1^ Weaning weight adjusted to 240 days of weaning age. ^2^ Annual average forage allowance.

**Table 3:** Default values of parameters used in the case study simulation.

Parameter ^1^	Default Value	Unit ^2^
Forage and Grazing Submodel		
Hay ME	1.7	Mcal/kg DM
Amount of hay provided	75	% DMI
Supplement to heifers ^3^	2	Kg DM/d
Length of supplementation ^3^	161	days
Management Submodel		
Cow-per-bull ratio	30	dml
Replacement rate	30	%
Birth rate	83	%
Calf death rate	6	%
Animal Nutrient Requirement and Dry Matter Intake Submodel		
Cow BCS	5	dml
Distance_flat_^4^	2000	m/days
Distance_sloped_^4^	0	m/days
Position	6	number/day
Standing	12	h/day

^1^ ME = metabolizable energy; BCS = body condition score. ^2^ DM = dry matter; DMI = DM intake. ^3^ For first-calf heifers and yearling heifers, as dried distillers’ grain cubes, digestible energy (DE) of 3.8 Mcal/kg DM. ^4^ Distance_flat_ = distance traveled daily; Distance_sloped_ = distance traveled daily as any vertical movement; Position = number of standing and lying changes (unit); Standing = time standing (hours).

## Data Availability

The original contributions presented in this study are included in the article/Supplementary Material. Further inquiries can be directed to the corresponding authors.

## Supplementary Materials

The following supporting information can be downloaded at: https://www.mdpi.com/article/10.3390/ani16111675/s1, Table S1: Emission factors from feeds and supplements; Table S2: Assumptions to simulate financial inputs and outputs; Table S3: Monthly satellite-derived standing forage mass from 2017 to 2023 in Texas A&M University McGregor Research Center (McGregor, TX, USA); Table S4: Observed versus predicted weaning weight from 2017 to 2022 in Texas A&M University McGregor Research Center (McGregor, TX, USA); Table S5: Average weather variables from McGregor, TX, USA; Table S6: Mean values and standard deviations of stochastic inputs used in the Monte Carlo simulation; Table S7: Spearman correlation coefficients of Monte Carlo simulation.

## Abbreviations

The following abbreviations are used in this manuscript:

Adj_AD_	Adjustment factor of predicted DMI for feed additive
Ad_jBF_	Adjustment factor of predicted DMI for breed
Adj_FA_DMI_	Adjustment factor of predicted DMI for forage allowance
Adj_MF_	Adjustment factor of predicted DMI for mud
Adj_TF_	Adjustment factor of predicted DMI for temperature
BW	Body weight
CBW	Calf birth weight
CH_4_	Methane
CO_2_	Carbon dioxide
CO_2e_	Carbon dioxide equivalent
DE	Digestible energy
DM	Dry matter
DMI	Dry matter intake
DMI_for_	Forage DMI
DST	Decision-support tool
EF	Emission factor
FA	Forage allowance
FM	Forage mass
GHG	Greenhouse gas
ME	Metabolizable energy
MEI	ME intake
ML	Machine learning
MY	Milk yield
N_2_O	Nitrous oxide
NEl	Net energy requirement for lactation
NEg	Net energy requirement for growth
NEm	Net energy requirement for maintenance
NEpr	Net energy requirement for pregnancy
OM	Organic matter
RNS	Ruminant Nutrition System
SD	Standard deviation
WW	Weaning weight

## Appendix A

### Appendix A.1. Energy Requirement for Maintenance

NEm_basal_ = (α_1_ × L × COMP × SEX + α_2_) × SBW^0.75^ (A1)

α_2_ = [(88.426 − 0.785 × T_p_ + 0.0116 × T_p_^2^) − 77]/1000 (A2)

COMP = 0.8 + 0.05 × (BCS[1–9] − 1) (A3)

NEm_act_ = (0.1 × Standing + 0.062 × Position + 0.621 × Distance_flat_ + 6.69 × Distance_sloped_) × BW/1000 (A4)

NEm = NEm_basal_ + NEm_act_ (A5)

MEm = NEm/k_m_ (A6)

where NEm_basal_ = the net energy requirement for basal metabolism (Mcal/d) [18]; COMP = adjustment for the previous plane of nutrition (dimensionless); SEX = effect of bulls (1.15) on α_1_ (dimensionless) [18]; α_2_ = adjustment for previous temperature (Mcal/kg^0.75^/d) [18], α_1_ = fasting metabolism: 0.070 Mcal/kg^0.75^/d for Bos taurus beef cows; 0.064 Mcal/kg^0.75^/d for Bos indicus [18], L = lactation factor (dimensionless); T_p_ = previous temperature (°C) [18]; BCS = body condition score; NEm_act_ = net energy requirement for physical activity (Mcal/d) [18]; Distance_flat_ = distance traveled daily (km); Distance_sloped_ = distance traveled daily as any vertical movement (km); Position = number of daily standing and lying changes (number); Standing = daily time standing (hours); NEm = net energy requirement for maintenance (Mcal/d); MEm = metabolizable energy requirement for maintenance (Mcal/d); and k_m_ = partial efficiency of use ME for maintenance, estimated based on the ratio between the net and metabolizable energy of diet [4,18].

### Appendix A.2. Energy Requirement for Pregnancy

NEpr = [(CBW/41) × 69.73 × (0.0323 − 0.000055 × t) × e^(0.0323 × t − 0.0000275 × t2)^]/1000 (A7)

MEpr = NEpr/k_pr_ (A8)

where CBW = calf birth weight (kg), t = time of pregnancy (days), NEpr = net energy requirements for pregnancy (Mcal/d), MEpr = metabolizable energy requirements for pregnancy (Mcal/d), and k_pr_ = partial efficiency of use ME for pregnancy, assumed as 13%.

### Appendix A.3. Energy Requirement for Lactation

MY = NEl/(0.0929 × MkF + 0.0547 MkTP/0.93 + 0.0395 × MkL) (A9)

NEl = MEI × k_lac_ (A10)

MElr = MEI − MEm − Mepr (A11)

MEI = DMI × ME_diet_ (A12)

where NElr = net energy requirements for lactation (Mcal/d), MY = milk yield (kg/d), MkF = milk fat content (g/100 g, default 4.85%); MkL = milk lactose content (g/100 g, default 4.85%); MkTP = milk true protein content (g/100 g, default 4.85%); MElr = metabolizable energy requirements for lactation (Mcal/d); k_lac_ = partial efficiency of use ME for lactation, assumed to be 64.4% [18]; MEI = metabolizable energy intake (Mcal/d); DMI = dry matter intake (kg/d); and ME_diet_ = metabolizable energy of diet (Mcal/kg dry matter)

### Appendix A.4. Energy Requirement for Growth

BWG = (12.341 × EQEBW^−0.6837^ × NEg^0.9116^)/0.956 (A13)

EQEBW = 0.891 × EQBW (A14)

EQBW = (478 × BW)/MW (A15)

NEg = MEg × k_g_ (A16)

MEg = MEI − MEm (A17)

where BWG = daily body weight gain, EQEBW = equivalent empty body weight (kg), EQBW = equivalent body weight (kg), BW = body weight (kg), MW = mature weight (kg), NEg = net energy requirements for gain (Mcal/d), MEg = metabolizable energy requirements for gain (Mcal/d), k_g_ = partial efficiency of use ME for growth estimated based on the ratio between the net and metabolizable energy of diet for gain [4], and MEI = metabolizable energy intake (Mcal/d).
