# Supplementary material for: Developing a Decision-Support Tool to Improve the Performance and Sustainability of Cow–Calf Grazing Systems Using Satellite Remote Sensing and Mechanistic Nutrition Models
Source: Animals (Basel). 2026 May 30;16(11):1675. doi: 10.3390/ani16111675 (PMC13255633; doi:10.3390/ani16111675)
Supplement: Supplementary file 1 [file animals-16-01675-s001.zip › animals-4306173-supplementary.pdf]

Developing a decision-support tool to improve the performance and sustainability of cow-calf grazing systems using satellite remote sensing and mechanistic nutrition models

M. H. M. R. Fernandes, J. M. Adams, PAS, J. A. R. Fernandes, and L. O. Tedeschi, PAS

Supplementary Data 1

Table S1. Emission factors from feeds and supplements

Source	unit	Value	Reference
Mineral salt	kg·CO2e·kg-1	0.16	[24]
Dried distillers' grain cubes	kg·CO2e·kg-1	0.57	Feedprint [23]
Oat hay	kg·CO2e·kg-1	0.20	Feedprint [23]

Table S2. Assumptions to simulate financial inputs and outputs

Year	2017	2018	2019	2020	2021	2022	2023	Average (±SD)
Calf price[1], USD/CWT[2]	146.00	148.00	141.00	138.00	147.00	168.00	223.00	158.71±27.71
**Variable costs**								
Hay[1], USD/ton[3]	105.00	121.00	117.00	138.00	147.00	174.00	196.00	142.57±30.27
Salt & mineral[4], USD/ton	440.92	440.92	440.92	440.92	661.39	661.39	1433.00	645.64±335.53
Supplement[4], USD/ton	440.92	440.92	440.92	440.92	440.92	440.92	551.16	456.67±38.57
Veterinary supplies[4], USD/animal/year	7.00	7.00	7.00	10.00	10.00	12.00	15.00	9.71±2.81
Marketing[4], USD/animal/year	8.77	9.03	9.03	12.00	12.00	15.00	15.00	11.55±2.53
Fuel, lube, repairs[4], USD/animal/year	23.04	21.59	21.59	24.21	21.59	23.16	30.05	23.61±2.79
Labor[4], USD/animal/year	1.10	1.10	1.10	1.10	1.10	1.10	1.10	1.10
**Fixed costs**								
Pasture[4], USD/ha	29.65	29.65	29.65	29.65	29.65	29.65	29.65	29.65

[1] Price based on USDA-NASS [27]
[2] CWT = price per 100 pounds (lb) of body weight. 1lb = 0.454 kg.
[3] Ton = 1,000 kg
[4] Price from Texas A&M AgriLife Extension Agricultural Economics [26], for District 8 cow-calf native pasture operation

Table S3. Monthly satellite-derived standing forage mass from 2017 to 2023 in Texas A&M University McGregor Research Center (McGregor, Texas, USA)

Year	Month	S-2 Forage mass[1] (g/m^2)	RAP cover[2] (%)	RAP ANPP[3] (g/m^2)	Forage mass[4] (g/m^2)
2017	1	132.01	62.82	5.60	86.45
2017	2	129.89	62.82	19.50	93.85
2017	3	120.13	62.82	34.79	97.32
2017	4	219.68	62.82	56.14	173.27
2017	5	213.02	62.82	51.06	165.89
2017	6	157.92	62.82	51.59	131.61
2017	7	160.08	62.82	48.40	130.96
2017	8	160.09	62.82	41.20	126.45
2017	9	137.78	62.82	37.94	110.39
2017	10	153.72	62.82	26.77	113.38
2017	11	125.48	62.82	8.22	83.99
2017	12	131.21	62.82	15.78	92.34
2018	1	131.21	62.82	2.56	84.04
2018	2	131.21	62.82	11.04	89.36
2018	3	143.12	62.82	22.13	103.81
2018	4	165.97	62.82	42.22	130.79
2018	5	151.30	62.82	47.44	124.85
2018	6	212.00	62.82	37.69	156.86
2018	7	122.59	62.82	24.07	92.13
2018	8	122.10	62.82	19.51	88.96
2018	9	131.67	62.82	22.23	96.68
2018	10	157.24	62.82	22.79	113.10
2018	11	137.94	62.82	9.41	92.56
2018	12	137.45	62.82	18.71	98.10
2019	1	233.71	61.75	5.64	147.80
2019	2	247.37	61.75	14.64	161.79
2019	3	269.75	61.75	23.84	181.30
2019	4	294.89	61.75	44.49	209.56
2019	5	308.74	61.75	48.15	220.38
2019	6	222.62	61.75	43.02	164.03
2019	7	285.90	61.75	44.42	203.98
2019	8	235.74	61.75	30.51	164.41
2019	9	208.28	61.75	22.66	142.61
2019	10	142.17	61.75	16.20	97.79
2019	11	144.56	61.75	4.40	91.99
2019	12	136.81	61.75	12.74	92.34

2020	1	127.32	62.99	5.27	83.52
2020	2	190.19	62.99	13.88	128.55
2020	3	271.14	62.99	26.15	187.26
2020	4	287.04	62.99	46.97	210.39
2020	5	275.79	62.99	49.66	205.00
2020	6	238.71	62.99	42.86	177.36
2020	7	244.24	62.99	35.54	176.23
2020	8	167.36	62.99	27.01	122.44
2020	9	186.81	62.99	34.38	139.32
2020	10	191.12	62.99	28.60	138.40
2020	11	164.69	62.99	9.17	109.51
2020	12	109.89	62.99	15.83	79.19
2021	1	141.32	54.21	5.88	79.80
2021	2	201.13	54.21	13.39	116.29
2021	3	177.98	54.21	30.94	113.26
2021	4	240.49	54.21	48.39	156.60
2021	5	292.59	54.21	53.23	187.47
2021	6	281.63	54.21	61.48	186.00
2021	7	265.09	54.21	57.55	174.91
2021	8	265.01	54.21	52.27	172.00
2021	9	209.73	54.21	40.87	135.85
2021	10	164.98	54.21	32.24	106.91
2021	11	159.91	54.21	10.07	92.15
2021	12	126.05	54.21	19.99	79.17
2022	1	88.30	72.75	4.09	67.22
2022	2	154.83	72.75	8.84	119.07
2022	3	149.04	72.75	21.38	123.98
2022	4	217.71	72.75	47.34	192.83
2022	5	285.54	72.75	48.63	243.11
2022	6	223.43	72.75	33.63	187.01
2022	7	209.99	72.75	21.59	168.48
2022	8	226.64	72.75	24.50	182.70
2022	9	148.94	72.75	31.50	131.27
2022	10	137.51	72.75	20.86	115.21
2022	11	194.38	72.75	9.13	148.05
2022	12	192.35	72.75	19.36	154.02
2023	1	230.06	55.80	7.44	132.52
2023	2	269.31	55.80	16.05	159.23
2023	3	330.76	55.80	37.71	205.61
2023	4	346.81	55.80	54.39	223.87
2023	5	341.33	55.80	58.39	223.05
2023	6	293.57	55.80	48.92	191.11
2023	7	228.55	55.80	29.58	144.03

2023	8	235.51	55.80	17.47	141.16
2023	9	148.89	55.80	29.21	99.38
2023	10	241.08	55.80	35.40	154.27
2023	11	243.41	55.80	11.25	142.10
2023	12	251.53	55.80	23.10	153.25

[1]Monthly satellite-derived forage mass based on Sentinel-2 satellite [20] and provided by Sigfarm Intelligence LLC (www.sigfarm .com)
[2] Percentage of abundance and distribution of herbaceous and annual herbaceous plants (10-m vegetation cover) from Rangeland Analysis Platform (https://rangelands.app/)
[3] Monthly forage growth based on 16-day aboveground biomass production from Rangeland Analysis Platform (https://rangelands.app/)
[4] Final forage mass = (S-2 Forage mass × RAP cover/100) + Forage mass

Table S4. Observed versus predicted weaning weight from 2017 to 2022 in Texas A&M University McGregor Research Center (McGregor, Texas, USA)

Year	Cows	WW obs.	WW pred.	FA_{min}
2017	794	249.6	243.9	2.45
2018	838	223.3	223.7	2.85
2019	889	221.2	223.4	3.6
2020	965	215.9	215.8	3.6
2021	1037	230.9	232.7	2.8
2022	1006	227.7	228.6	3.1

WW = weaning weight, obs = observed, pred = predicted, FA_{min} = minimum FA of the grazing ecosystem to input in Equation 3.

Table S5. Average weather variables from McGregor, Texas, USA[1].

Month	Temperature (°C)	Least night temperature (°C)	Relative humidity (%)	Wind speed (km/h)	HRS[2] (h)
January	14.44	1.11	70	15.77	10
February	16.11	3.33	70	16.73	10
March	20.00	7.22	70	17.21	10
April	24.44	11.67	70	16.89	10
May	28.33	16.67	70	15.44	10
June	32.22	20.56	70	13.84	10
July	35.00	22.22	70	13.03	10
August	35.00	22.22	70	12.39	10
September	31.67	18.33	70	11.42	10
October	26.11	12.78	70	13.84	10
November	20.00	7.78	70	14.96	10
December	14.44	2.22	70	14.96	10

[1]Source: Weather Underground, https://www.wunderground.com
[2] Hours exposed to direct sunlight.

Table S6. Mean values and standard deviations of stochastic inputs used in the Monte Carlo simulation[1].

Input variable[2]	Mean	Standard deviation
Cow BW, kg	550	55
Pregnancy rate (%)	83	8.3
Calf price, USD/CWT	158.71	27.71
Hay, USD/ton	142.57	30.27
Dry FM – January, g/m^2	97.33	27.99
Dry FM - February, g/m^2	124.02	26.41
Dry FM - March, g/m^2	144.64	41.74
Dry FM - April, g/m^2	185.33	30.94
Dry FM - May, g/m^2	195.67	37.16
Dry FM - June, g/m^2	170.57	19.73
Dry FM - July, g/m^2	155.82	33.96
Dry FM - August, g/m^2	142.59	30.46
Dry FM - September, g/m^2	122.21	18.09
Dry FM - October, g/m^2	119.86	18.09
Dry FM - November, g/m^2	108.62	24.17
Dry FM - December, g/m^2	106.91	30.25
FA_{min}, kg dry FM/kg BW	3.0	0.3
FA_{max}, kg dry FM/kg BW	4.5	0.45

[1] Conducted using the @Risk® software v8.2 (Palisade Corp., Ithaca, NY)
[2] BW= body weight, FM = forage mass, FA_{min} = minimum FA of the grazing ecosystem, FA_{max} = maximum FA of the grazing ecosystem, CWT = price per 100 pounds (lb) of body weight. 1lb = 0.454 kg, Ton = 1,000 kg,

Table S7. Spearman correlation coefficients of Monte Carlo simulation[1].

Input variable[2]	Weaning Weight	Net Return
Cow BW, kg	0.90	0.39
Pregnancy rate (%)	.	0.40
Calf price, USD/CWT	.	0.65
Hay, USD/ton	.	-0.14
Dry FM – January, g/m^2	0.30	0.25
Dry FM - February, g/m^2	-0.01	0.23
Dry FM - March, g/m^2	-0.05	.
Dry FM - April, g/m^2	0.15	.
Dry FM - May, g/m^2	0.03	0.06
Dry FM - June, g/m^2	0.20	0.17
Dry FM - July, g/m^2	-0.16	.
Dry FM - August, g/m^2	0.06	.
Dry FM - September, g/m^2	-0.18	-0.30
Dry FM - October, g/m^2	-0.24	0.11

Dry FM - November, g/m^2	.	.
Dry FM - December, g/m^2	-0.05	.
FA_{min}, kg dry FM/kg BW	-0.17	0.16
FA_{max}, kg dry FM/kg BW	-0.21	.

[1] Conducted using the @Risk® software v8.2 (Palisade Corp., Ithaca, NY)

References

20. Fernandes, M.H.M.R.; Fernandes Junior, J.S.; Adams, J.M.; Lee, M.; Reis, R.A.; Tedeschi, L.O. Using sentinel-2 satellite images and machine learning algorithms to predict tropical pasture forage mass, crude protein, and fiber content. *Sci. Rep.* **2024**, *14*, 8704. https://doi.org/10.1038/s41598-024-59160-x

23. Vellinga, T.V.; Blonk, H.; Marinussen, M.; van Zeist, W.J.; de Boer, I.J.M.; Starmans, D. *Methodology Used in Feedprint: A Tool Quantifying Greenhouse Gas Emissions of Feed Production and Utilization*; Livestock Research, Report 674; Wageningen UR Livestock Research: Wageningen, The Netherlands, 2013; p. 121.

24. Cardoso, A.S.; Berndt, A.; Leytem, A.; Alves, B.J.R.; de Carvalho, I.d.N.O.; de Barros Soares, L.H.; Urquiaga, S.; Boddey, R.M. Impact of the intensification of beef production in Brazil on greenhouse gas emissions and land use. *Agric. Syst.* **2016**, *143*, 86–96. https://doi.org/10.1016/j.agsy.2015.12.007.

26. AGECOEXT. Texas A&M AgriLife Extension Service. Department of Agricultural Economics. Beef Cow-Calf Standardized Performance Analysis and Budgets. 2025. Available online: https://agecoext.tamu.edu/resources/budgets/ (accessed on 19 October 2025).

27. U.S. Department of Agriculture, National Agricultural Statistics Service. (USDA-NASS). Available online: https://quickstats.nass.usda.gov/ (accessed on 19 July 2025)
